# Clinical Assessment of Abrocitinib, Tofacitinib, and Cyclosporine in Adult Patients With Moderate to Severe Atopic Dermatitis: A Retrospective Analysis

**DOI:** 10.7759/cureus.85448

**Published:** 2025-06-06

**Authors:** Shahnawaz Bashir

**Affiliations:** 1 Dermatology, Dermis Skin Laser and Hair Transplant Centre, Srinagar, IND

**Keywords:** abrocitinib, atopic dermatitis (ad), cyclosporine, from india, jak inhibitors, real world, retrospective cohort, tofacitinib

## Abstract

Background: Moderate to severe atopic dermatitis (M2S AD) is a chronic, relapsing inflammatory skin disease characterized by intense pruritus and eczematous lesions with significant impact on quality of life. Conventional therapies like cyclosporine are commonly prescribed drugs, along with tofacitinib as an off-label use. Recently, abrocitinib received approval for the management of M2S AD, a selective JAK1 inhibitor that has been well studied in the Western population. However, real-world comparative data among cyclosporine, tofacitinib, and abrocitinib remain limited. Therefore, we conducted a retrospective single-center study to assess the effectiveness and safety of cyclosporine, tofacitinib, and abrocitinib in adult patients with M2S AD over a 16-week treatment period.

Materials and methods: A single-center retrospective cohort study was conducted using electronic medical records of adult M2S AD patients treated with abrocitinib 100 mg once a day, tofacitinib 5 mg twice a day, or cyclosporine 100 mg once a day from April 2024 to March 2025. Clinical assessments were analyzed at baseline and weeks 4, 8, 12, and 16 using Eczema Area and Severity Index (EASI), Scoring Atopic Dermatitis (SCORAD), and Dermatology Life Quality Index (DLQI). Serum IgE levels and adverse events (AEs) were also analyzed.

Results: Fifteen patients (five per group) were included as per the inclusion and exclusion criteria. At week 16, all three groups showed significant within-group improvements in EASI, SCORAD, DLQI, and Numerical Rating Scale (NRS) scores (p<0.001). However, compared with tofacitinib and cyclosporine, abrocitinib showed greater reduction in mean scores (EASI: 87% vs. 64% and 51%; SCORAD: 93% vs. 55% and 40%; NRS: 85% vs. 62% and 51%; all p<0.01), respectively. DLQI also demonstrated a similar trend in abrocitinib (80%) against tofacitinib (59%) and cyclosporine (47%) (p<0.01). A significant decrease was also observed for serum IgE level in the abrocitinib group. In the abrocitinib group, two patients had headaches and nausea each, whereas two patients reported infection in the tofacitinib group. In the cyclosporine group, two patients had hypertension. All AEs were mild and resolved over the study period, with no treatment discontinuations. There was no statistical difference between any of the groups.

Conclusions: In our small sample-sized analysis, all treatment groups were found to be efficacious and safe. However, abrocitinib showed a better clinical response than the other two groups in achieving symptom control. However, due to the small sample size, these claims can’t be generalized, and further large-scale studies are needed to validate these findings and assess long-term outcomes.

## Introduction

Atopic dermatitis (AD) is a chronic, relapsing inflammatory skin disease characterized by intense pruritus, which often worsens at night, along with xerosis, lichenification, and skin thickening [1]. It substantially affects health-related quality of life, disrupts sleep, and can contribute to psychological distress [2,3]. Given its widespread impact on patients and their families, comprehensive evaluation, including patient-reported outcomes (PROs), has become an essential component of disease management [4,5].

Successful long-term control of AD requires effective communication between clinicians and patients, incorporating both objective clinical assessments and subjective experiences [6]. In clinical practice, objective tools such as the Eczema Area and Severity Index (EASI), Scoring Atopic Dermatitis (SCORAD), and Investigator’s Global Assessment (IGA) are widely used. However, PRO instruments such as the Dermatology Life Quality Index (DLQI) and the Patient-Oriented Eczema Measure (POEM) are underutilized due to their perceived complexity and time constraints [2].

Moderate to severe AD (M2S AD) remains particularly challenging to manage in terms of severity of disease, higher relapse rate, and lack of a uniform treatment algorithm [7]. International and US-based guidelines recommend systemic treatments, including Janus kinase (JAK) inhibitors (abrocitinib, baricitinib, and upadacitinib) and biologic therapies such as dupilumab and tralokinumab, with conventional therapies like cyclosporine recommended conditionally [8-11].

Cyclosporine continues to be a commonly prescribed agent, although newer therapies like tofacitinib as an off-label indication have shown promising real-world efficacy [12]. However, many patients either do not respond adequately or experience adverse events (AEs) [13]. Abrocitinib, a selective JAK1 inhibitor recently approved in India, has demonstrated positive outcomes in M2S AD [14]. However, real-world comparative data among cyclosporine, tofacitinib, and abrocitinib remain limited. We selected these agents based on commonly prescribed molecules in a real-world scenario and excluded dupilumab due to its restricted access.

This retrospective, exploratory study aimed to descriptively assess the clinical effectiveness and safety of abrocitinib, tofacitinib (off-label), and cyclosporine (off-label) in a small cohort of adult patients with M2S AD treated at a single center in India over a 16-week period.

## Materials and methods

After receiving approval from the Good Society for Ethical Research (approval number: GSER/2025/BMR/CL/093), we performed a retrospective cohort study using electronic medical records from our dermatology clinic. We included adult patients diagnosed with M2S AD (EASI score ≥16 [15] and SCORAD ≥25 [16]) who were treated with one of the following oral systemic agents between April 2024 and March 2025: cyclosporine (100 mg once daily), tofacitinib (5 mg twice daily), or abrocitinib (100 mg once daily).

To ensure data completeness and reliability, only patients with documented clinical assessments at baseline and weeks 4, 8, 12, and 16 were included. All included patients had completed the full 16-week treatment period without significant modifications to the prescribed dosing regimen. Before the start of treatment with either of these drugs, a washout period of at least four weeks was considered for patients who had been using previous therapies for the management. Patients were excluded if they discontinued or switched therapy during the treatment period or if they were receiving concomitant systemic immunosuppressants, biologics, topical medications, or phototherapy. Only moisturizers were considered concomitant medications.

Patient demographics, comorbidities, disease profile, and previous therapeutic strategies were extracted from the electronic medical records. The effectiveness of either of the drugs was evaluated in terms of improvement in EASI, SCORAD, Numerical Rating Scale (NRS), and DLQI at weeks 4, 8, 12, and 16, compared with baseline. Our primary endpoint was the percentage reduction in EASI and SCORAD scores from baseline at each follow-up visit. In particular, we analyzed the change in mean score of each parameter and also the percentage reduction in mean score at the end of therapy compared to baseline. EASI 75 was not analyzed due to the small sample size in each group. Additionally, we analyze the mean change in SCORAD. Regarding PRO measures, which were our secondary endpoints, we analyzed the reduction in mean change in NRS at week 16 compared to baseline in each group. The change in quality of life was analyzed using the DLQI as reported by patients at each visit. In addition, serum IgE levels were also analyzed at baseline and the end of treatment.

Regarding safety, the occurrence of any AEs as reported by patients during the entire study duration, focusing on treatment-emergent AEs, severe AEs, and AEs leading to either discontinuation of the drug or dose modification, was reported.

Statistical analysis

Statistical analyses were performed using SPSS Statistics version 18.0 (IBM Corp. Released 2009. IBM SPSS Statistics for Windows, Version 18.0. Armonk, NY: IBM Corp.). Continuous variables were expressed as means ± standard deviation, and categorical variables as frequencies and percentages. The Shapiro-Wilk test was used to assess the normality of continuous variables (EASI, SCORAD, DLQI, NRS). For normally distributed data, intergroup differences at week 16 were analyzed using one-way analysis of variance (ANOVA), followed by Tukey’s post-hoc test for pairwise comparisons. Non-normally distributed data were assessed using the Kruskal-Wallis test, followed by Dunn’s post-hoc test. Intragroup comparisons (baseline vs. week 16) were performed using paired t-tests for parametric data and the Wilcoxon signed-rank test for non-parametric data. Since multiple outcomes were tested (EASI, SCORAD, DLQI, NRS), Bonferroni correction was applied to control for type I error. AE frequencies between groups were compared using the chi-square test, with Fisher’s exact test applied when expected frequencies were <5. A two-sided p-value <0.05 was considered statistically significant.

## Results

After screening the data of 28 patients (abrocitinib, 5; cyclosporine, 12; and tofacitinib, 10), we analyzed the data of 15 patients who were prescribed either abrocitinib 100 mg once daily, tofacitinib 5 mg twice daily, or cyclosporine 100 mg once daily in M2S AD. Thirteen patients’ data were not considered as per the inclusion and exclusion criteria (Table 1).

**Table 1:** Reasons for exclusion from analysis TCS: topical corticosteroids

Treatment group	No. of patients	Reason for exclusion
Abrocitinib	1	Expensive treatment, patient discontinued after one month of treatment
Tofacitinib	3	Not effective as monotherapy, topical corticosteroids added
	2	Lost to follow-up
Cyclosporine	5	Lost to follow-up
	1	Excluded due to concomitant therapy (TCS)
	1	Discontinued due to early response

Most patients (60%) in each group were male. Most of the patients had previously received some form of anti-AD treatment (details couldn’t be extracted). Demographic details are depicted in Table 2.

**Table 2:** Demographic details at baseline One-way ANOVA used for continuous variables; chi-square test for categorical data (gender). All p>0.05 indicate no statistically significant differences at baseline. SD: standard deviation, EASI: Eczema Area and Severity Index, SCORAD: Scoring Atopic Dermatitis, DLQI: Dermatology Life Quality Index, NRS: Numerical Rating Scale, IgE: immunoglobulin E, ANOVA: analysis of variance

Variable; (mean ± SD)	Abrocitinib	Tofacitinib	Cyclosporine	p-value
N	5	5	5	-
Age (years)	32.8 ± 4.1	34.2 ± 3.8	33.4 ± 4.0	0.88
Disease duration (years)	6.75 ± 2.36			
Male (%)	60%	60%	60%	1
EASI	25.3 ± 2.8	25.7 ± 4.3	25.4 ± 3.1	0.98
SCORAD	60.7 ± 2.7	60.2 ± 5.4	60.6 ± 3.2	0.97
DLQI	20.2 ± 7.4	20.7 ± 2.6	20.4 ± 1.8	0.98
NRS	8.4 ± 1.2	8.2 ± 1.7	8.6 ± 2.5	0.94
Serum IgE (IU/ml)	1250 ± 130	1245 ± 125	1230 ± 110	0.96

Efficacy assessment

Under the primary endpoint, the mean EASI score of abrocitinib 25.3 ± 2.8 reduced to 15.2 ± 3.2, 10.4 ± 2.2, 5.2 ± 3.3, and 3.2 ± 1.2 at weeks 4, 8, 12, and 16, respectively. This reduction was statistically significant at all time intervals compared to baseline. Similar findings were observed for the tofacitinib and cyclosporine groups, as shown in Table 3. On intergroup comparison, abrocitinib was found to be statistically significant compared to the other two groups (p<0.01).

**Table 3:** Reduction in mean scores at different time intervals in all three groups Intergroup comparisons were conducted using one-way ANOVA with Tukey’s post-hoc test. All p<0.01 and p<0.001 indicate statistically significant differences among the three treatment groups. EASI: Eczema Area and Severity Index, SCORAD: Scoring Atopic Dermatitis, DLQI: Dermatology Life Quality Index, NRS: Numerical Rating Scale, ANOVA: analysis of variance

						p-value	Intergroup p-value at week 16		
Parameter	Treatment group	4 weeks	8 weeks	12 weeks	16 weeks	Intragroup	Abrocitinib vs. tofacitinib	Abrocitinib vs. cyclosporine	Tofacitinib vs. cyclosporine
EASI	Abrocitinib	15.2 ± 3.2	10.4 ± 2.2	5.2 ± 3.3	3.2 ± 1.2	<0.001	0.0005	0.0004	0.1
	Tofacitinib	19.2 ± 2.7	14.7 ± 1.4	10.3 ± 2.6	9.2 ± 2.1				
	Cyclosporine	20.6 ± 2.4	18.6 ± 2.3	15.3 ± 3.1	12.4 ± 3.3				
SCORAD	Abrocitinib	43.4 ± 3.1	30.3 ± 1.7	20.2 ± 1.2	4.2 ± 1.6	<0.001	0.0001	0.0001	0.002
	Tofacitinib	45.1 ± 2.7	38.7 ± 3.5	32.3 ± 2.4	27.2 ± 1.1				
	Cyclosporine	50.3 ± 1.8	47.2 ± 2.3	40.2 ± 1.7	36.5 ± 2.3				
DLQI	Abrocitinib	14.2 ± 1.1	9.2 ± 1.4	4.9 ± 1.1	4.1 ± 1.1	<0.01	0.001	0.001	0.15
	Tofacitinib	17.4 ± 2.5	13.4 ± 1.2	10.7 ± 2.2	8.5 ± 1.8				
	Cyclosporine	18.7 ± 1.1	15.6 ± 2.4	12.8 ± 3.1	10.8 ± 2.7				
NRS	Abrocitinib	4.5 ± 2.2	2.7 ± 1.1	1.8 ± 1.1	1.3 ± 1.1	<0.001	0.03	0.004	0.16
	Tofacitinib	5.7 ± 1.1	4.3 ± 1.3	3.3 ± 1.2	3.1 ± 1.1				
	Cyclosporine	6.8 ± 2.7	5.4 ± 1.2	4.3 ± 1.1	4.2 ± 1.2				

**Figure 1:**
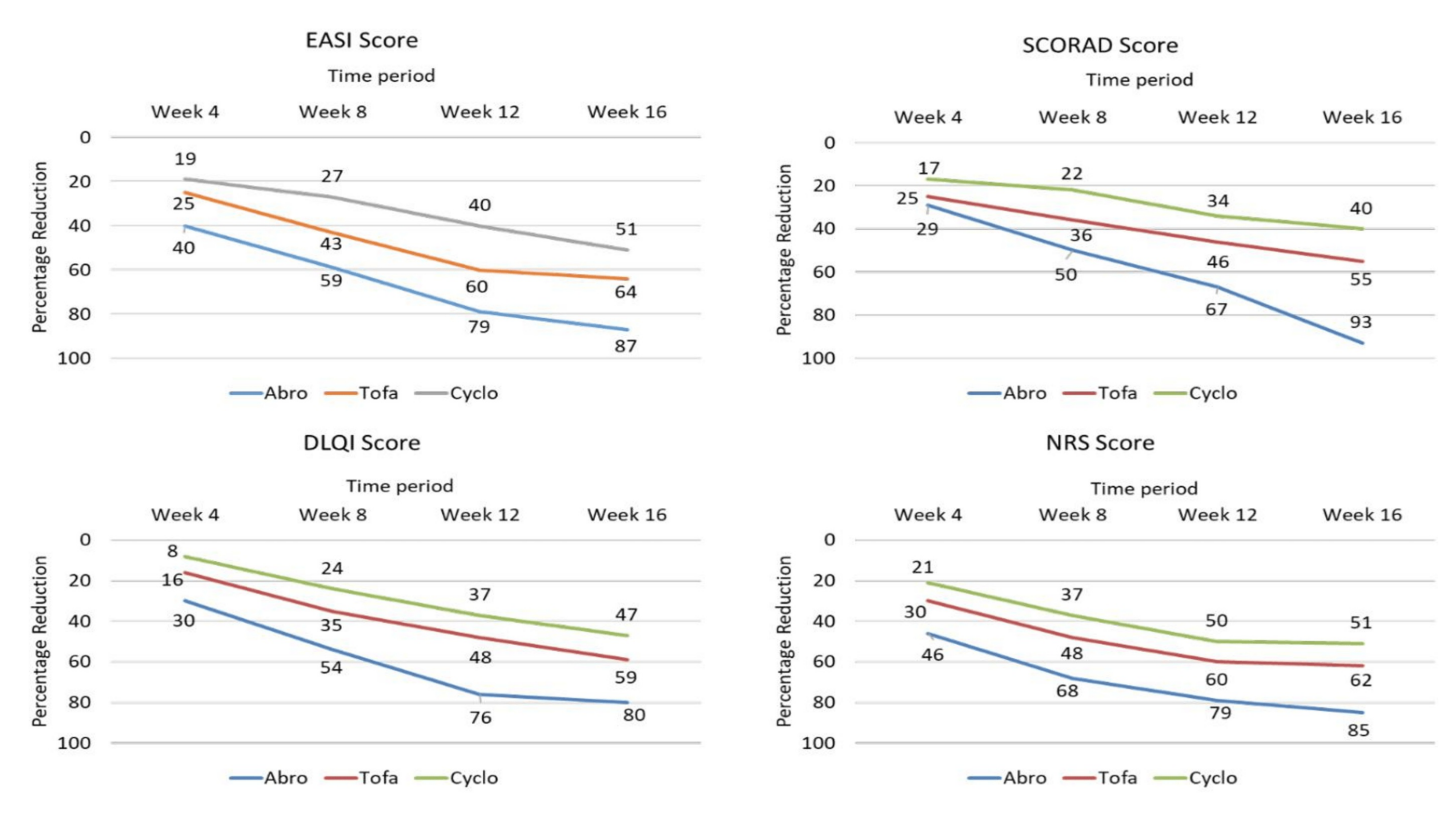
Percentage reduction in mean scores at different time intervals in all groups EASI: Eczema Area Severity Score, SCORAD: Scoring Atopic Dermatitis, DLQI: Dermatology Life Quality Index, NRS: Numerical Rating Scale, Abro: abrocitinib, Tofa: Tofacitinib, Cyclo: Cyclosporine

Similarly, for SCORAD, mean scores of 60.7 ± 2.7, 60.2 ± 5.4, and 60.6 ± 3.2 at baseline reduced to 4.2 ± 1.6, 27.2 ± 1.1, and 36.5 ± 2.3 at week 16 for abrocitinib, tofacitinib, and cyclosporine, respectively. Although reductions were statistically significant in all groups from baseline, an intergroup comparison revealed that abrocitinib was statistically superior to the other two groups (p < 0.01). Additionally, the mean NRS scores for itch and improvement in quality of life, as measured by the DLQI, showed significant results for abrocitinib, as shown in Table 3. The percent change reduction in all groups is depicted in Figure 1.

Additionally, the serum IgE level of 1250 ± 130 IU/ml at baseline reduced to 410 ± 65 at week 16 in the abrocitinib group. Similar findings were noted for the tofacitinib and cyclosporine groups, as shown in Table 4. These findings were statistically significant in all groups at week 16 compared to baseline. On intergroup comparison, abrocitinib was found to be statistically significant compared to the other two groups. However, when compared to tofacitinib and cyclosporine, no statistically significant difference was noted.

**Table 4:** Reduction in mean IgE levels at week 16 in all three groups Intergroup comparisons were conducted using one-way ANOVA followed by Tukey’s post-hoc test. Statistically significant differences were noted between abrocitinib vs. cyclosporine (p=0.009) and abrocitinib vs. tofacitinib (p=0.007). No significant difference was found between cyclosporine vs. tofacitinib (p=0.61). IgE: Immunoglobulin E, ANOVA: analysis of variance

Parameter	Abrocitinib (n=5)	Cyclosporine (n=5)	Tofacitinib (n=5)
Baseline serum IgE (IU/mL)	1250 ± 130	1230 ± 110	1245 ± 125
Serum IgE at week 16 (IU/mL)	410 ± 65	710 ± 90	770 ± 85
Absolute reduction (IU/mL)	840 ± 85	520 ± 95	475 ± 88
Percentage reduction (%)	67.30%	42.30%	38.20%
Intragroup significance (p-value)	p=0.003	p=0.015	p=0.022

Safety assessment

Abrocitinib was well tolerated, with only two AEs in two patients: headache and nausea. Both AEs were mild in nature and transient, resolving throughout the period. Tofacitinib was associated with a slightly higher risk of infection, including increased incidence of zoster and opportunistic infections (such as upper respiratory infections), in two patients. In contrast, the cyclosporine group had two patients with hypertension. All these AEs were mild, and no additional treatment was required. Also, there was no discontinuation of any of the drugs.

## Discussion

To the best of our knowledge, this is the first clinical assessment evaluating the effectiveness and safety of abrocitinib, tofacitinib, and cyclosporine in patients with M2S AD in India.

AD is increasingly recognized as a heterogeneous, immune-mediated disorder primarily driven by type 2 inflammation, with additional contributions from Th17 and Th22 pathways in specific populations [17]. This evolving understanding of immune-pathological interactions has driven a shift from broad-spectrum immunosuppressants, such as cyclosporine, to targeted therapies like JAK inhibitors, which disrupt intracellular cytokine signaling pivotal to disease activity [18].

In our study, abrocitinib, a selective JAK1 inhibitor, demonstrated better improvements across all clinical measures -SCORAD, EASI, DLQI, and NRS - compared to the other two groups. Its targeted inhibition of IL-4, IL-13, IL-31, and thymic stromal lymphopoietin, cytokines implicated in pruritus and barrier dysfunction, likely underpins its superior efficacy [19]. In contrast, tofacitinib, a pan-JAK inhibitor, also acts on JAK2 and JAK3, leading to broader immunosuppression but potentially less focused suppression of type 2 cytokines, which may explain its relatively diminished performance.

While cyclosporine remains a conventional systemic treatment, its clinical effect in this study was modest and slower to onset, consistent with its mechanism of action, which involves the inhibition of calcineurin and the indirect suppression of cytokine transcription [20]. Adverse effects, including hypertension and nephrotoxicity observed in two patients, further highlight long-standing safety concerns associated with cyclosporine in chronic AD management [21].

Abrocitinib produced significant clinical improvements by week 4, with efficacy comparable to findings from the JADE trials, where over 50% EASI reduction was observed by week 2 [22]. In our study, there was an 87% reduction in mean EASI score with abrocitinib at week 16, outperforming the tofacitinib (-64%) and cyclosporine (-51%) groups. These findings align with real-world data from the BIODAY registry, where abrocitinib achieved a 71% reduction in EASI at week 16, with 53% of patients reaching EASI-75 [23].

In addition to objective improvements, abrocitinib also demonstrated a significant impact on PROs, particularly in reducing pruritus and enhancing quality of life. At week 16, patients on abrocitinib reported an 85% reduction in itch scores, substantially higher than the 41% reduction observed in the BIODAY registry [23]. This rapid itch relief is likely due to IL-31 inhibition via JAK1 targeting, which has been shown to modulate itch signaling at the level of the dorsal root ganglia [24].

Notably, abrocitinib also induced better reduction in serum IgE levels, with a mean decrease of 67.3% (p=0.003), significantly outperforming both cyclosporine and tofacitinib (p=0.009 and p=0.007, respectively). As elevated IgE levels are associated with disease severity and immune dysregulation in AD, this reinforces abrocitinib’s targeted action on the type 2 inflammatory axis [25].

Historically, much of the efficacy data on cyclosporine in AD have come from studies conducted before standardized tools, such as EASI, were widely adopted [26]. In a more recent study involving 356 patients receiving long-term cyclosporine, nearly 50% discontinued treatment due to lack of efficacy or intolerable side effects [27], underscoring the need for better-tolerated and more effective options.

While tofacitinib outperformed cyclosporine in this study concerning SCORAD only, it did not match abrocitinib in terms of efficacy or speed of response. This may reflect its broader JAK inhibition profile, leading to non-selective immunosuppression and potentially limiting its utility in chronic dermatological conditions. The emerging consensus supports the use of selective JAK1 inhibition, as it offers a favorable balance between efficacy and safety in the treatment of inflammatory skin diseases [28].

## Conclusions

All treatment groups achieved good symptom control by the end of 16 weeks in patients with M2S AD. However, abrocitinib showed a better response to treatment as compared to the other two groups, particularly in patients requiring rapid symptomatic relief and meaningful improvements in quality of life. We also want to point out that abrocitinib is the only drug approved for the management of M2S AD in India for a longer duration. Hence, owing to its better clinical response, significant reduction in itch and IgE levels, and favorable safety profile as seen in this study, it can be considered as a preferred molecule in the armamentarium of M2S AD management.

Although this is the first real-world clinical assessment, it has several limitations, including a small sample population, a short observation period, a lack of observation of remission periods, and a need for long-term safety and effectiveness studies. Due to the retrospective nature, some of the confounding factors could not be ruled out. While the results are encouraging, they can’t be generalized and warrant validation through larger prospective observational studies and cost-effectiveness analyses to establish the long-term role of abrocitinib in routine dermatologic practice.
